# Preservation of the Remains of Extinct Species

**Published:** 1865

**Authors:** 


PRESERVATION OF THE REMAINS OF EXTINCT SPECIES.—"Almost all the hard parts of animals—the bones and so on—are composed chiefly of phosphate of lime and carbonate of lime. Some years ago, I had to make an inquiry into the nature of some very curious fossils sent me from the north of Scotland. Fossils are usually hard, bony structures that have become imbedded in the way I have described, and have gradually acquired the nature and solidity of the body with which they are associated; but in this case I had a series of *holes* in some pieces of rock, and nothing else. Those holes, however, had a certain definite shape about them, and when I got a skilful workman to make castings of the interior of these holes, I found that they were the impressions of the joints of a back bone and of the armor of a great reptile, twelve or more feet long. This great beast had died and got buried in the sand; the sand had gradually hardened over the bones, but remained porous. Water had trickled through it, and that water being probably charged with a superfluity of carbonic acid, had dissolved all the phosphate and carbonate of lime, and the bones themselves had thus decayed and entirely disappeared; but as the sandstone happened to have consolidated by that time, the precise shape of the bones was retained. If that sandstone had remained soft a little longer, we should have known nothing whatsoever of the existence of the reptile whose bones it had incased.

"How certain it is that a vast number of animals which have existed at one period on this earth have entirely perished, and left no trace whatever of their forms, may be proved to you by other considerations. There are large tracts of sandstone in various parts of the world, in which nobody has yet found anything but footsteps. Not a bone of any description, but an enormous number of traces of footsteps. There is no question about them. There is a whole valley in Connecticut covered with these footsteps, and not a single fragment of the animals which made them has yet been found. Let me mention another case, while upon that matter, which is even more surprising than those to which I have yet referred. There is a limestone formation near Oxford, at a place called Stonesfield, which had yielded the remains of certain very interesting mammalian animals, and up to this time, if I recollect rightly, there have been found seven specimens of its lower jaws, and not a bit of anything else, neither limb bones or skull, or any part whatever; not a fragment of the whole system! Of course it would be preposterous to imagine that the beasts had nothing else but a lower jaw! The probability is, as Dr. Buckland showed, as the result of his observations on dead dogs in the River Thames, that the lower jaw, not being secured by very firm ligaments to the bones of the head, and being a weighty affair, would easily be knocked off, or might drop away from the body as it floated in water in a state of decomposition. The jaw would thus be deposited immediately, while the rest of the body would float and drift away altogether, ultimately reaching the sea, and perhaps becoming destroyed. The jaw becomes covered up and preserved in the river silt, and thus it comes that we have such a curious circumstance as that of the lower jaws in the Stonesfield slates. So that, you see, faulty as these layers of stone in the earth's crust are defective as they necessarily are as a record, the account of contemporaneous vital phenomena presented by them is, by the necessity of the case, infinitely more defective and fragmentary."—(*Huxley.*)

